# Why bubble-free electrosurgical settings with continuous low-pressure saline perfusion are particularly suitable for duodenal endoscopic submucosal dissection

**DOI:** 10.1055/a-2882-8426

**Published:** 2026-06-15

**Authors:** Masafumi Kitamura, Takashi Ueno, Kosei Hashimoto, Hisashi Fukuda, Yuji Ino, Tomonori Yano, Hironori Yamamoto

**Affiliations:** 1 Department of medicine, Division of gastroenterology 12838 Jichi Medical UniversityShimotsukeTochigiJapan; 2 Department of Endoscopic Research and International Education Funded by FUJIFILM Medical Co., Ltd 12838 Jichi Medical UniversityShimotsukeTochigi PrefectureJapan

Duodenal endoscopic submucosal dissection (ESD) is technically demanding. This difficulty is primarily attributable to poor endoscopic maneuverability, extremely thin submucosal and muscular layers, and abundant submucosal vessels. In particular, once bleeding occurs during submucosal dissection, bleeding rapidly obscures visualization and may lead to submucosal hematoma formation, increasing procedural difficulty and perforation risk.

We previously reported a bubble-free strategy for saline-immersion ESD that combines optimized electrosurgical settings with continuous low-pressure saline perfusion (CLPSP). 1​ ​2​ A key feature of this strategy is the use of softCOAG, which delivers a low-voltage continuous current without spark generation, allowing pre-coagulation of submucosal vessels without producing bubbles under saline immersion ( Fig. 1 ). This allows stable and smooth submucosal dissection without the need for hemostatic forceps.

**Fig. 1:**
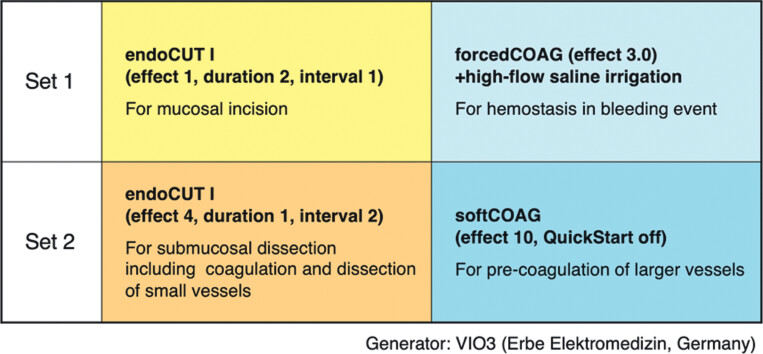
Optimized electrosurgical settings for the bubble-free strategy, enabling effective bleeding control.

This strategy is particularly advantageous in duodenal ESD. Minimizing the use of hemostatic forceps allows effective hemostasis with a limited thermal coagulation area, thereby reducing thermal injury to the extremely thin duodenal muscular layer, potentially lowering the risk of delayed perforation ( Fig. 2 ). Moreover, even when bleeding occurs during dissection, immediate hemostasis can be achieved using the same dissection device, without device exchange, maintaining a clear visual field and preventing hematoma formation ( Fig. 3 ). Furthermore, maintaining the intestinal lumen in a collapsed state under CLPSP helps prevent further thinning of the already extremely thin duodenal submucosal layer, facilitating safer and more stable dissection.

**Fig. 2:**
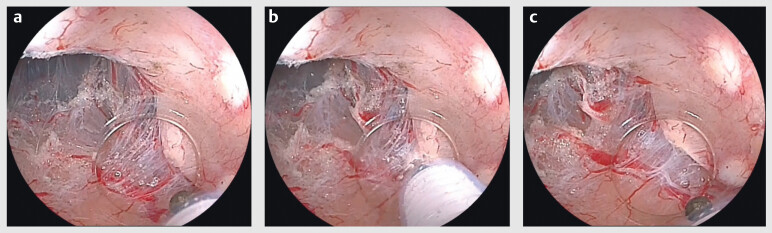
Precise knife-tip pre-coagulation in a thin submucosal space, minimizing thermal injury to the muscular layer. ( **a** ) A submucosal vessel in thin submucosal space. ( **b** ) Pre-coagulation using softCOAG with the knife tip. ( **c** ) Pre-coagulation achieved while minimizing thermal injury to the muscular layer.

**Fig. 3:**
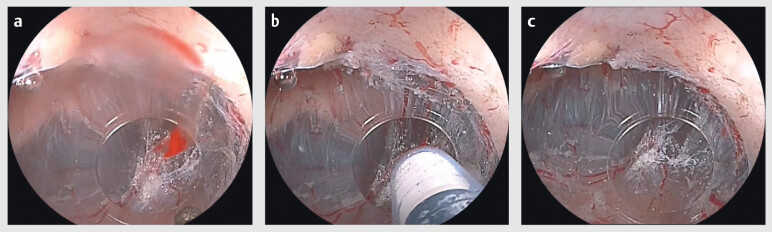
Immediate hemostasis achieved with the knife tip in softCOAG without device exchange, maintaining a clear visual field and preventing hematoma formation. ( **a** ) Active bleeding. ( **b** ) Coagulation using softCOAG (effect 10, QuickStart off) with the knife tip. ( **c** ) Immediate hemostasis preventing hematoma formation.

Balloon-assisted ESD using a short-type double-balloon endoscope can further improve endoscopic positioning and maneuverability in the deep duodenum. 3​ In addition, the double-balloon overtube also provides a practical drainage route for CLPSP, enabling stable saline perfusion during the procedure. 4​

In combination, balloon-assisted ESD using bubble-free electrosurgical settings with CLPSP represents a strategy particularly suitable for duodenal ESD ( Fig. 4 ). This approach may improve endoscopic positioning and maneuverability, ensure continuous visualization, and allow effective bleeding control with minimal thermal damage ( Video 1 ).

**Fig. 4:**
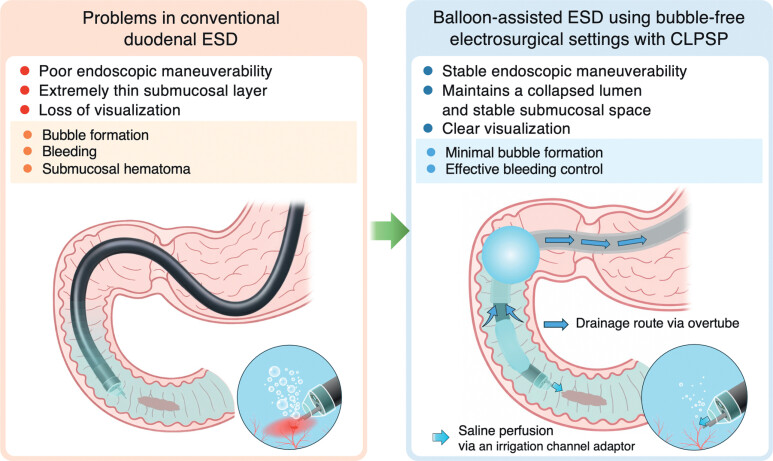
Conceptual comparison between conventional endoscopic submucosal dissection (ESD) and integrated balloon-assisted ESD using bubble-free electrosurgical settings with continuous low-pressure saline perfusion (CLPSP).

**Video 1** Balloon-assisted duodenal endoscopic submucosal dissection (ESD) using bubble-free electrosurgical settings with continuous low-pressure saline perfusion (CLPSP).


Endoscopy_UCTN_Code_TTT_1AO_2AG_3AD

## References

[1] Kitamura M Ino Y Hashimoto K A bubble-free strategy for saline-immersion endoscopic submucosal dissection: optimized electrosurgical settings and continuous low-pressure saline perfusion Endoscopy 2026 58 E180 E181 41633388 10.1055/a-2780-1100 PMC12867568

[2] Ino Y Fukuda H Ueno T Continuous low-pressure saline perfusion for gastric endoscopic submucosal dissection Endoscopy 2024 56 E880 E881 39424360 10.1055/a-2427-9429 PMC11489009

[3] Yamamoto H Miura Y Duodenal ESD: Conquering difficulties Gastrointest Endosc Clin N Am 2014 24 235 244 24679234 10.1016/j.giec.2013.11.007

[4] Hashimoto K Yamamoto H Despott E J The overtube in balloon-assisted endoscopic submucosal dissection is useful for improving maneuverability and as the drainage route in the continuous low-pressure saline-perfusion method VideoGIE 2025 10 599 601 41262197 10.1016/j.vgie.2025.06.007 PMC12624532

